# Impact of preterm birth on maternal well-being and women's perceptions of their baby: a population-based survey

**DOI:** 10.1136/bmjopen-2016-012676

**Published:** 2016-10-10

**Authors:** Jane Henderson, Claire Carson, Maggie Redshaw

**Affiliations:** Policy Research Unit in Maternal Health and Care, National Perinatal Epidemiology Unit, Nuffield Department of Population Health, University of Oxford, Oxford, UK

**Keywords:** preterm birth, maternal health, anxiety, perceptions, postnatal health

## Abstract

**Background:**

Approximately 15 million babies were born preterm worldwide in 2010 and in England in 2014 there were 52 249 preterm births. Preterm babies are at increased risk of poor outcomes and this can put enormous strain on the family.

**Objective:**

This study aimed to test the hypothesis that giving birth preterm affects maternal health, mood and well-being, and alters women's feelings and perceptions about their baby.

**Methods:**

Data collected in a population-based survey of maternity care in England in 2014 were used. Women were randomly selected and asked about their pregnancy, birth and postnatal experience when their babies were about 3 months of age. Descriptive statistics were produced, and logistic regression used to estimate ORs, adjusted for key confounders.

Main outcome measures—Women's self-reported postnatal health, Edinburgh Postnatal Depression Scale, women's perceptions of their baby.

**Results:**

4578 women returned completed questionnaires. Of these, 42 (0.9%) had babies born before 32 weeks' gestation and 243 (5.5%) at 32–36 weeks. Comparing the three gestational age groups, no statistically significant differences in rates of depressive symptoms measured on the Edinburgh Postnatal Depression Scale were found. However, using a health problems checklist, anxiety, fatigue and flash-backs were more common in mothers of preterm babies. Overall, mothers of preterm babies had less early contact with their baby, more postnatal health problems, substantially less positive feelings towards their baby and made less use of the support options available.

**Conclusions:**

Women with preterm births are at increased risk of ill-health and negative feelings about their baby in the early months after birth. They make less use of postnatal services and support than other women and this may be an area where the use of specialist services would be appropriate.

**Strengths and limitations of this study**

- This is a large population-based study using a sample of births in England for which the responses were generally well completed with a low proportion of missing data (generally <3%).
- The proportion of babies born preterm is close to that of the England and Wales population for the same year.
- A limitation of the study is the response rate of 47%, which is common with surveys of women who have recently given birth, with some under-representation of women who were young, unmarried, living in deprived areas and women born outside the UK.
- The items relating to antenatal and postnatal health were based on a symptom checklist, however, the Edinburgh Postnatal Depression Scale as a standard measure was also used.
- All data were collected at 3 months post-partum, and this cross-sectional survey design means it is difficult to attribute causality to the observed associations. Potential recall bias should be considered, as the women who experienced a preterm birth may examine their care more critically. However, for key variables there is good evidence for effective recall of salient events around childbirth.

## Introduction

Data from the Office of National Statistics (ONS) indicate that delivery at <37 weeks' gestation occurred in 7.5% of births in England and Wales in 2014, and at <32 weeks' gestation in 1.2% of births, with a total of 52 249 babies born preterm in England.^1^ Worldwide in 2010, it is estimated that ∼15 million babies were born preterm and more than 1 million died as a direct result of their prematurity.^2^

Babies born preterm are at increased risk of a range of poor outcomes including respiratory distress syndrome, necrotising enterocolitis and neonatal sepsis.^2^ ^3^ In the long-term, they are more likely to experience motor and sensory impairment, delay in cognitive development and behavioural problems than babies born at term.^3^ Some studies have found that specialist programmes improve outcomes^4^ although the evidence is mixed.^5^

A preterm birth can put enormous strain on the family, particularly if the baby is seriously ill.^6^ ^7^ The neonatal unit is an unfamiliar environment in which parents can feel lost and frustrated.^6^ The effects of a preterm birth on parental stress are exacerbated by caesarean section and by either no or limited contact with their baby soon after birth.^6^ In studies largely based on single sites/hospitals the risk of anxiety, depression, post-traumatic stress disorder (PTSD) and poorer overall well-being have been documented as significantly increased in parents of preterm babies, with these ill effects reported to persist for a considerable time especially following very preterm birth.^6^ ^8–13^

Qualitative research in this area using focus groups of parents and health professionals identified the themes of ‘shattered expectations’, ‘helplessness and horror’, ‘the infant's precarious health’, ‘prolonged uncertainty’ and the need to foster ‘adaptation to the birth and care of a preterm infant’.^3^ They focused on the impact of altered parental roles and the importance of health professionals encouraging breastfeeding, early physical contact (kangaroo care) and family-centred practices to restore parental agency and facilitate the reconstruction of parental roles.^3^

The present study aimed to test the hypothesis that giving birth preterm adversely affects maternal mood and well-being, and influences women's feelings and perceptions of their baby.

## Methods

This study used data collected as part of a national survey of women's experiences of maternity care in England.^14^ The survey, in the form of a self-completion postal questionnaire, was sent to 10 000 women randomly selected from birth registrations by the Office for National Statistics (ONS) in January 2014. Women who were aged <16 years, and those whose baby had died, were excluded. The questionnaire asked about care during pregnancy, labour and birth and the postnatal period, about sociodemographic characteristics, and neonatal care if applicable. It was sent when the baby was 12 weeks of age with tailored reminders sent to non-respondents after 2 weeks, 6 weeks and 10 weeks following the initial questionnaire. Further details of the survey are given in the survey report.^14^

Women reported gestational age at delivery which, after exclusion of anomalous values (10 in total), was aggregated into three groups: very preterm babies <32 weeks, preterm babies 32–36 weeks and term babies of 37 or more weeks' gestation (including post-term). Women were asked about their own health and well-being in the postnatal period using the Edinburgh Postnatal Depression Scale (EPDS) 3 months after the birth, and also completed a checklist of 13 symptoms that could have been experienced at 10 days, 1 month and 3 months postnatally. The checklist items included anxiety, depression, fatigue and symptoms of PTSD including sleep problems not related to the baby, flash-backs to the labour or birth, and difficulties in concentrating. These had been used in previous National Maternity Surveys 14 and were selected for analysis based on the literature suggesting such adverse effects may occur following preterm birth. 6 8–13 With regard to early contact and the development of the mother–infant relationship, women were asked whether they had been able to hold their baby, have skin-to-skin contact and breastfeed soon after birth, and when they first felt that their baby really belonged to them with six answer options ranging from ‘during pregnancy’ to ‘not quite yet’. They were also asked about their perceptions of their baby currently (at around 3 months) using a predefined checklist of 16 adjectives of which half were positive and half negative, and whether they felt that their baby was more or less difficult than average.^15^ Women were also asked about their postnatal care and whether they had used a range of possible sources of support, advice or information, such as a parent support group, a drop-in clinic or a parenting website. In the UK women are likely to see a midwife in the immediate postpartum period and may receive home visits. Following this, care is then the responsibility of an area-based health visitor, a health professional (commonly a nurse or midwife) who focuses on infant and to some extent maternal health in the early years. Preterm infants are likely to have specialist paediatric follow-up.

The association between preterm birth and maternal mental health was assessed first in univariate analysis comparing the three gestational age groups using χ^2^, second using logistic regression to estimate ORs, with the various health outcomes analysed in turn as the dependent variable. Where health outcomes were significant in univariate analysis, they were entered into the logistic regression.

ORs were adjusted for confounding by parity, maternal age, black or minority ethnic group (BME) and index of multiple deprivation. It was hypothesised that women with a pre-existing health problem or pregnancy-related complication may have been better prepared for a preterm birth and therefore this group was analysed separately. All analyses were carried out in Stata (V.13.1), using the 5% level to determine statistical significance. Return of the questionnaire was taken as informed consent.

## Results

In total, 4578 women returned completed questionnaires representing a 47% response rate (after exclusion of undeliverable questionnaires from the denominator). Questionnaires were returned at (mean and median) 15 weeks postpartum. Younger, unmarried women, those living in deprived areas and women who were born outside the UK were significantly less likely to respond to the survey (χ^2^ p<0.05). Nevertheless, 16% of the respondents were from BME groups, 24% were born outside the UK and 13% did not have a partner at the time of the survey. Gestational age at birth was reported by 4461 women (97.4%). Of these, 42 (0.9%) delivered very preterm (<32 weeks), 243 (5.5%) moderately preterm (32–36 weeks) and 4176 (93.6%) were term deliveries (37 or more weeks' gestation). The sociodemographic characteristics of the three groups are shown in table 1. Overall, there was no significant difference between the groups by index of multiple deprivation, single motherhood or ethnicity, but very preterm babies were significantly more likely to be born to women who were primiparous and those who had left full-time education aged <17 years. No clinical or medical record data were available in this survey-based study, including information on the ultrasound (US) methods used to estimate gestational age, however, almost all women in the study sample reported having first trimester US scans (95%) and later anomaly scans (99%). As would be expected, prematurity was highly associated with both birthweight and multiplicity of birth. Similarly, there was a strong inverse correlation between gestational age at birth and admission to, and duration of stay, in a neonatal unit. Two-thirds of mothers of very preterm babies stayed in the hospital following their own discharge to be with their baby.

**Table 1:** Sociodemographic characteristics of term, preterm and very preterm population

	Gestation at birth							
	<32 weeks		32–36 weeks		37 or more weeks		Total	
	Number	Per cent	Number	Per cent	Number	Per cent	Number	Per cent
Median (IQR) maternal age	33.5 (28, 37.5)		31 (28, 35)		31 (27, 35)		31 (27, 35)	
Index of multiple deprivation								
1 (least deprived)	9	21.4	45	18.5	822	19.7	876	19.6
2	7	16.7	42	17.3	797	19.1	846	19.0
3	7	16.7	50	20.6	858	20.6	915	20.5
4	12	28.6	53	21.8	897	21.5	962	21.6
5 (most deprived)	7	16.7	53	21.8	801	19.2	861	19.3
Black or minority ethnic group	5	12.5	46	19.5	638	15.7	689	15.9
Born in the UK	37	88.1	183	75.3	3184	76.3	3404	76.3
Primiparous*	21	55.3	137	58.8	2001	49.4	2159	49.9
Left full-time education aged <17 years*	9	23.1	54	22.5	666	16.2	729	16.6
Single mother	7	16.7	33	13.6	519	12.4	559	12.5
Birthweight <2500 g**	38	95.0	133	57.1	158	3.9	329	7.5
Multiple birth**	4	9.5	34	14.0	36	0.9	74	1.6
Admitted to neonatal unit**	39	100.0	143	62.4	352	9.4	534	13.3
Median (IQR) duration in neonatal unit (days)	49 (28, 74)		11 (3, 20)		2.1 (0.3, 6)		4 (1, 12)	
Still in neonatal unit at time of the survey**	4	10.3	2	1.4	6	1.7	12	2.2
Mothers of NNU babies stayed in hospital after discharge**	24	63.2	82	59.4	136	43.2	242	49.3
Mode of delivery**								
Normal vaginal	22	66.7	99	47.8	2456	61.5	2577	60.9
Instrumental	0	0.0	37	17.9	614	15.4	651	15.4
Planned caesarean	5	15.2	39	18.8	421	10.5	465	11.0
Unplanned caesarean	6	18.2	32	15.5	504	12.6	542	12.8
Soon after birth mother able to…								
Hold baby**	7	16.7	171	71.5	3745	90.9	3923	89.1
Skin-to-skin**	5	11.9	153	63.7	3597	87.3	3755	85.3
Breastfeed**	4	9.5	117	50.4	3130	76.5	3251	74.5

*p<0.05 **p<0.01.

IQR, inter-quartile range; NNU, neonatal unit.

Mothers of preterm and very preterm babies were significantly less likely to have a normal vaginal delivery and more likely to have both planned and unplanned caesarean births. They were also significantly less likely to be able to hold, have skin-to-skin contact and breastfeed soon after birth compared to mothers of term babies (table 1). In the majority of cases this was because of the baby's condition, although in some cases the mother was too unwell and in a few cases it was not offered (data not shown).

Mothers of preterm (but not very preterm) babies were significantly more likely to have long-term health problems (such as diabetes or epilepsy) complicating the pregnancy (table 2). Mothers of both preterm and very preterm babies were also significantly more likely to have pregnancy-specific problems (such as hypertension or placental problems). There was no significant difference in the proportion of women who felt well in the first few days after giving birth or at the time of the survey (about 3 months), but very preterm babies were substantially more likely to still have health problems at the time of the survey.

**Table 2:** Proportion of mothers and babies with health problems by gestation at birth

	Gestation at birth							
	<32 weeks		32–36 weeks		37 or more weeks		Total	
	Number	Per cent	Number	Per cent	Number	Per cent	Number	Per cent
Chronic health problems complicating pregnancy**								
	3	5.7	38	15.8	346	8.4	387	8.8
Pregnancy-specific problems**								
	26	52.0	108	45.6	1059	25.9	1193	27.3
Mother physically well in first few days								
	16	39.0	100	41.7	1879	45.6	1995	45.3
Mother physically well at time of survey								
	32	78.0	213	89.1	3636	89	3881	88.9
Baby health problems at time of survey**								
	18	45.0	46	19.5	525	12.9	589	13.5
EPDS >11 at 3 months	7	18.4	30	13.6	433	11.3	470	11.5
Depression/blues								
10 days	9	22.0	94	39.0	1437	34.7	1540	34.8
1 month	4	9.8	31	12.9	636	15.4	671	15.2
3 months	1	2.4	11	4.6	275	6.6	287	6.5
Fatigue								
10 days	17	41.5	95	39.4	1621	39.2	1733	39.2
1 month*	17	41.5	78	32.4	1112	26.9	1207	27.3
3 months**	11	26.8	32	13.3	467	11.3	510	11.5
Anxiety								
10 days**	17	41.5	55	22.8	789	19.1	861	19.5
1 month	8	19.5	27	11.2	481	11.6	516	11.7
3 months	6	14.6	13	5.4	240	5.8	259	5.9
Flash-backs								
10 days	7	17.1	31	12.9	468	11.3	506	11.4
1 month	5	12.2	24	10.0	273	6.6	302	6.8
3 months**	6	14.6	12	5.0	147	3.6	165	3.7
Sleep problems not related to the baby								
10 days	3	7.3	14	5.8	239	5.8	256	5.8
1 month	3	7.3	14	5.8	200	4.8	217	4.9
3 months	1	2.4	5	2.1	175	4.2	181	4.1
Difficulties in concentrating								
10 days	8	19.5	39	16.2	649	15.7	696	15.7
1 month	8	19.5	30	12.4	563	13.6	601	13.6
3 months	3	7.3	15	6.2	349	8.4	367	8.3

* p<0.05 **p<0.01 EPDS Edinburgh Postnatal Depression Scale.

The specific problems that were reported by mothers varied by time period (table 2). At 10 days, there was a significantly higher rate of anxiety in mothers with the most preterm infants, compared to higher gestational age groups. A similar pattern was observed at 1 month and 3 months, although the difference was no longer statistically significant. Overall 11.5% of women experienced depressive symptoms, however, a marked gradient was observed across the gestational age groups in the proportion of women who experienced symptoms of depression as indicated by the EPDS score at 3 months (18.4% of mothers of very preterm babies, 13.6% of the moderately preterm and 11.5% of mothers of term babies). The association was not statistically significant at the 5% level, but given the small numbers in these groups, statistical significance should be interpreted with caution. This pattern was not apparent in self-reported depression at 1 and 3 months, although rates were substantially lower.

At 1 month mothers of both preterm and very preterm babies reported more fatigue, and at 3 months flash-backs and fatigue were significantly more common in mothers of very preterm babies. It appears that flashbacks (a PTSD-type symptom) are more persistent in mothers of preterm babies.

The proportion of mothers reporting flashbacks declined in all the gestational age groups over the first 3 months of their baby's life. However, this reduction was slower in the most preterm group, so that by 3 months postpartum there was a significant difference in the proportion of women who still experienced them.

Mothers' reports of their feelings about their baby are shown in table 3. There was a significant difference in when mothers of preterm and very preterm babies felt their baby really belonged to them with 5% of mothers of very preterm babies responding ‘not quite yet’ in relation to their babies who were just over 3 months old, compared to 0.6% of mothers of term babies. Mothers of preterm and very preterm babies also used fewer positive adjectives to describe their baby at this time, such as ‘happy’ and ‘responsive’, and mothers of very preterm babies used more negative adjectives about their baby, such as ‘demanding’ and ‘grizzly’. Similarly, they were substantially more likely to consider their baby ‘more difficult than average’ (table 3). However, women who had depressive symptoms at this time (EPDS score >11) were significantly more likely to have more negative perceptions of their baby (p<0.01).

**Table 3:** Mothers' feelings about the baby at the time of the survey

	Gestation at birth							
	<32 weeks		32–36 weeks		37 or more weeks		Total	
	Number	Per cent	Number	Per cent	Number	Per cent	Number	Per cent
When mother first felt that baby belonged**								
During pregnancy	15	38.5	110	47.0	2217	54.4	2342	53.9
Immediately after birth	0	0.0	45	19.2	887	21.8	932	21.4
First few days	3	7.7	25	10.7	430	10.6	458	10.5
First few weeks	8	20.5	35	15.0	360	8.8	403	9.3
Only recently	11	28.2	14	6.0	156	3.8	181	4.2
Not quite yet	2	5.1	5	2.1	24	0.6	31	0.7
Number of positive adjectives used by mother about baby**								
1–4	27	64.3	102	42.0	1221	29.2	1350	30.3
5–6	9	21.4	83	34.2	1636	39.2	1728	38.7
7 or more	6	14.3	58	23.9	1319	31.6	1383	31
Number of negative adjectives used by mother about baby**								
0	16	38.1	66	27.2	847	20.3	929	20.8
1	12	28.6	117	48.1	2209	52.9	2338	52.4
2 or more	14	33.3	60	24.7	1120	26.8	1194	26.8
Baby felt to be more or less difficult than average*								
More difficult	5	12.5	13	5.5	151	3.7	169	3.9
Average	25	62.5	127	53.6	2173	53.4	2325	53.5
Easier	10	25.0	97	40.9	1748	42.9	1855	42.7

*p<0.05 **p<0.01.

Women also reported on their postnatal care following hospital discharge (table 4). Mothers of very preterm babies were significantly less likely to be visited at home by a midwife or have phone contact, generally because their baby was still in hospital. When they did see a midwife, women had somewhat less confidence and trust in them and would not have wanted to see them more frequently. Mothers of both preterm and very preterm babies were less likely to use all types of postnatal support, significantly so with respect to drop-in clinics, peer support and parenting websites (table 4).

**Table 4:** Postnatal care of women following term and preterm birth

	Gestation at birth							
	<32 weeks		32–36 weeks		37 or more weeks		Total	
	Number	Per cent	Number	Per cent	Number	Per cent	Number	Per cent
Woman visited at home by midwife**								
Yes	25	64.1	217	91.9	4027	97.7	4269	97.1
Saw midwife in clinic	4	10.3	8	3.4	69	1.7	81	1.8
Not offered visit	5	12.8	4	1.7	9	0.2	18	0.4
Moved house	1	2.6	0	0.0	0	0.0	1	0.0
No, other reason†	4	10.3	7	3.0	18	0.4	29	0.7
Woman had confidence and trust in midwives seen after going home								
Always	18	69.2	143	65.0	2815	68.8	2976	68.6
Sometimes	5	19.2	62	28.2	1102	26.9	1169	27.0
Rarely	1	3.8	9	4.1	124	3.0	134	3.1
Never	2	7.7	6	2.7	49	1.2	57	1.3
Woman would have liked to have seen postnatal midwives…*								
More often	6	18.2	57	25.1	946	23.3	1009	23.4
Less often	2	6.1	14	6.2	202	5.0	218	5.1
Saw as much as wanted	25	75.8	156	68.7	2906	71.7	3087	71.6
Since birth, used…								
Baby clinic	8	19.0	69	28.4	1273	30.5	1350	30.3
Drop-in clinic**	5	11.9	72	29.6	1507	36.1	1584	35.5
Children's centre**	6	14.3	84	34.6	1551	37.1	1641	36.8
Parents' group	6	14.3	31	12.8	452	10.8	489	11.0
Peer support	10	23.8	62	25.5	1336	32.0	1408	31.6
Postnatal classes	1	2.4	15	6.2	264	6.3	280	6.3
Baby café	1	2.4	8	3.3	227	5.4	236	5.3
Online support	9	21.4	67	27.6	1189	28.5	1265	28.4
Parenting website*	11	26.2	70	28.8	1545	37.0	1626	36.4

*p<0.05 **p<0.01.

†Mainly included women whose baby was still in hospital.

Table 5 shows the results of logistic regression on maternal health. After adjustment for parity, maternal age, BME group and index of multiple deprivation, postpartum mothers of very preterm babies were significantly more likely to suffer from anxiety at 10 days, fatigue and flash-backs at 3 months and at 3 months feel that their baby belonged to them only recently or not quite yet, and that their baby was more difficult than average. Mothers of preterm babies born at 32–36 weeks did not have a statistically significant increase in any adverse outcomes. Analyses of data on women who had health problems or pregnancy complications produced very similar findings (table 5) although the CIs were wider due to a smaller sample size.

**Table 5:** Adjusted ORs (95% CI) of effects of preterm and very preterm birth on maternal outcomes for all women, and restricted to women with health problems or pregnancy complications, compared to women with term birth

	All women			Women with health/pregnancy complications		
	<32 weeks	32–36 weeks	37 or more weeks	<32 weeks	32–36 weeks	37 or more weeks
	Adjusted OR† (95% CI)	Adjusted OR† (95% CI)	Adjusted OR† (95% CI)	Adjusted OR† (95% CI)	Adjusted OR† (95% CI)	Adjusted OR† (95% CI)
Anxiety at 10 days	2.67 (1.36 to 5.23)**	1.23 (0.90 to 1.70)	1.00	2.16 (0.92 to 5.08)	1.12 (0.72 to 1.73)	1.00
Fatigue at 1 month	1.81 (0.92 to 3.54)	1.29 (0.97 to 1.73)	1.00	2.13 (0.91 to 4.97)	0.83 (0.55 to 1.27)	1.00
Fatigue at 3 months	2.52 (1.20 to 5.30)*	1.27 (0.86 to 1.88)	1.00	2.60 (1.03 to 6.54)*	1.31 (0.78 to 2.20)	1.00
Flash-backs at 3 months	5.32 (2.16 to 13.10)**	1.39 (0.76 to 2.56)	1.00	5.52 (1.77 to 17.21)**	1.79 (0.82 to 3.93)	1.00
*At 3 months…*						
Feeling that the baby belonged: only recently or not quite yet	12.13 (5.75 to 25.59)**	1.61 (0.95 to 2.72)	1.00	8.99 (3.46 to 23.40)**	1.07 (0.50 to 2.31)	1.00
Number of negative adjectives used to describe baby: 2 or more	1.55 (0.79 to 3.04)	0.83 (0.61 to 1.14)	1.00	1.27 (0.53 to 3.03)	0.81 (0.53 to 1.24)	1.00
Baby considered: more difficult than average	3.97 (1.51 to 10.45)**	1.59 (0.88 to 2.85)	1.00	4.26 (1.20 to 15.14)*	2.33 (1.14 to 4.78)*	1.00

*p<0.05; **p<0.01.

†Adjusted for parity, black or minority ethnic group, maternal age and index of multiple deprivation.

When early interaction with the baby was included in the model (as binary Yes/No variables), holding and skin-to-skin contact were protective against anxiety, flash-backs and negative feelings about the baby but prematurity was no longer significantly associated with the outcome (data not shown). This suggests that prematurity and early interaction are highly associated; early interaction is only possible if the baby is not too unwell and not too preterm.

## Discussion

Pregnancy and childbirth are major life events with a potential to impact substantially on women's health and well-being. Preterm birth, with the complex associated events and experiences, contrasts markedly with birth at term and presents a challenge to parents in terms of immediate response and the longer term.^16^ ^17^ This study suggests that mothers of preterm and very preterm babies have more health problems both antenatally and during the early postnatal months, including significantly more anxiety, fatigue and flashbacks. The prevalence of depression based on EPDS score also appears higher in mothers of the most preterm babies (18.5% (95% CI: 8.9% to 34.1%)), compared to term (11.3% (10.3% to 12.3%)); although small numbers mean this finding must be interpreted with caution it is consistent with our other findings. The overall prevalence of depressive symptoms as assessed by EPDS was 11.5%, which is in line with what would be expected for the population and is similar to that reported in a large cohort 8 weeks and 8 months after birth using >12 cut-off.^18^ ^19^ However, self-reported depression in this study using a checklist was much lower and no trend was discernible across the gestational age groups. This difference between the measures may reflect mothers' perceptions of their own well-being; perhaps with low expectations of how they should be feeling at 3 months postpartum they to do not perceive their feelings to be abnormal or worthy of the label ‘depression’, and yet the symptoms captured by the EPDS suggest that they may be experiencing marked low mood.

Mothers of preterm babies have less early contact with their baby, as might have been expected and report substantially less positive feelings towards their baby in the early months. Their feelings that their baby was more difficult than average and their greater use of negative adjectives to describe their baby may reflect the fact that preterm babies tend to be more difficult to handle and interact with early on and that some still had health problems at the time of the survey.^20^ ^21^ The findings for this population agree with those reported in a study of 420 mothers of babies admitted to neonatal units.^22^ Also using an adjective checklist, mothers' perceptions of their baby were more negative if the baby was born at earlier gestations or required ventilatory support. Relationship building between parents and babies can take time and is not straightforward in the context of adversity that commonly includes separation and concern about future developmental outcomes.^23^ ^24^ It may be that women's more negative appraisal of their baby following preterm birth and in many instances an anxious pregnancy, affected the way they felt about their baby and adjusted to the developing parent–infant relationship. Some may have delayed their feelings of attachment in the sense of the baby ‘belonging’, and this psychological process and differences in investment, as well as real practical difficulties may be reflected in their responses and the significant association between such negative feelings and depression. However, further longitudinal prospective research would be required to explore these issues and possible causal mechanisms and pathways.

In the postnatal period, the women were less likely to see a community midwife, because of not being at home, and less likely to feel confidence and trust in that person, possibly because of the precarious health of the infant and having built up trust and reliance on the staff of the neonatal unit. A potentially important finding was that after discharge home mothers of preterm babies made significantly less use of the various support options available, such as drop-in clinics and online support. Possible explanations for this may relate to the infant's health, the mothers' feeling that a general drop-in clinic was not appropriate, they may still be receiving support from the hospital, the baby's time in special care is likely to have delayed the introduction to such groups, or the mother's own lack of postnatal well-being or confidence may be a barrier.

This study benefited from being a large, population-based sample and the questions were generally well completed with a low percentage of missing data (generally <3%). As all questionnaires were mailed out at the same time following the birth irrespective of gestation (not corrected age), the potentially wide range of time since birth was avoided. However, a limitation of the study is the response rate of 47%, which is common with surveys of such women.^25^ ^26^ There was significant under-representation of women who were young, unmarried, living in deprived areas and women born outside the UK, potentially resulting in bias. Nevertheless, the proportion of babies born very preterm, 0.9%, matched closely to that of the England and Wales population at 1.2%^1^ and was only a slight underestimate for preterm birth at 5.5% compared to 6.3% in the England and Wales population.^1^ In absolute terms, there are relatively few mothers in the preterm birth groups and resultant low power means that findings must be interpreted with caution. The cross-sectional survey design necessitated data collection at 3 months postpartum and it is thus difficult to attribute causality to the findings. For example, mothers of difficult babies are more likely to become depressed but also depressed mothers are more likely to describe their child as difficult.^13^ ^27^ Although causality cannot be inferred from cross-sectional studies, the fact that preterm birth precedes postnatal outcomes lends weight to our interpretation that preterm birth does affect how women feel and how they perceive their babies. However, recall bias may have led to women who had experienced a preterm birth examining their care more critically than other women and data could not be checked against independent medical records. However, key measures such as the EPDS and perceptions of their infant were reported at the same time as the survey return and recall of earlier salient events around childbirth, such as gestational age, is generally good.^28–30^ Over 95% of women had a dating scan in early pregnancy so their reports of gestational age are likely to be reasonably accurate. A further limitation was that the items relating to antenatal and postnatal health were based on a symptom checklist rather than validated measures, however, the EPDS as a standard measure was also used in collecting data on maternal well-being.

The findings of this study in relation to the mother being able to hold, have skin-to-skin contact with and breastfeed her baby soon after birth are consistent with those of other studies^6^ in that mothers of preterm babies were generally less able to interact with their baby soon after birth due to the health of their newborn at that time. The adverse effects on maternal health and feelings of attachment or connectedness with her baby are also consistent with other studies^6^ ^8^ ^10^ ^23^ ^31^ and are likely to relate to the loss of parenting role while the baby is in the neonatal unit.

Unsurprisingly, women with health-related and pregnancy-related problems were significantly more likely to have a preterm birth. It might have been expected that these women would have been better prepared for the experience than women who delivered early unexpectedly. However, maternal postnatal health in these groups was as similarly badly affected by a preterm birth as other groups.

While mothers with preterm infants made less use of routinely available postnatal services and support than other women, follow-up of preterm mothers as well as babies, at least in the short term, could be an area where after preterm birth targeted family-focused services would be appropriate and could contribute to improvements in maternal well-being.

## Conclusions

This study has shown that women who experience a preterm birth are at increased risk of ill-health and negative feelings about their baby in the early months with their baby. They make less use of postnatal services and support than other women and this may be an area where specialist services would be appropriate.

## References

[1] Office for National Statistics. Birth characteristics in England and Wales, 2014. Table 7 2015.

[2] McCormick MC, Litt JS, Smith VC Prematurity: an overview and public health implications. Annu Rev Public Health 2011;32:367–79. 10.1146/annurev-publhealth-090810-182459 21219170

[3] Lasiuk GC, Comeau T, Newburn-Cook C Unexpected: an interpretive description of parental traumas’ associated with preterm birth. BMC Pregnancy Childbirth 2013;13(Suppl 1):S13 10.1186/1471-2393-13-S1-S13 23445715 PMC3561145

[4] Robinson M, Israel C, Parker D Randomised trial of parental support for families with very preterm children. Arch Dis Child Fetal Neonatal Ed 1998;79:F4–F11. 10.1136/fn.79.1.F4 9797618 PMC1720827

[5] Johnson S, Ring W, Anderson P Randomised trial of parental support for families with very preterm children: outcome at 5 years. Arch Dis Child 2005;90:909–15. 10.1136/adc.2004.057620 15899921 PMC1720549

[6] Franck LS, Cox S, Allen A Measuring neonatal intensive care unit-related parental stress. J Adv Nurs 2005;49:608–15. 10.1111/j.1365-2648.2004.03336.x 15737221

[7] Muller-Nix C, Forcada-Guex M, Pierrehumbert B Prematurity, maternal stress and mother-child interactions. Early Hum Dev 2004;79:145–58. 10.1016/j.earlhumdev.2004.05.002 15324994

[8] Vigod SN, Villegas L, Dennis CL Prevalence and risk factors for postpartum depression among women with preterm and low-birth-weight infants: a systematic review. BJOG 2010;117:540–50. 10.1111/j.1471-0528.2009.02493.x 20121831

[9] Treyvaud K, Lee KJ, Doyle LW Very preterm birth influences parental mental health and family outcomes seven years after birth. J Pediatr 2014;164:515–21. 10.1016/j.jpeds.2013.11.001 24359937 PMC3950307

[10] Gray PH, Edwards DM, O'Callaghan MJ Parenting stress in mothers of very preterm infants—influence of development, temperament and maternal depression. Early Hum Dev 2013;89:625–9. 10.1016/j.earlhumdev.2013.04.005 23669559

[11] Eiser C, Eiser JR, Mayhew AG Parenting the premature infant: balancing vulnerability and quality of life. J Child Psychol Psychiatry 2005;46:1169–77. 10.1111/j.1469-7610.2005.00415.x 16238664

[12] Treyvaud K, Ure A, Doyle LW Psychiatric outcomes at age seven for very preterm children: rates and predictors. J Child Psychol Psychiatry 2013;54:772–9. 10.1111/jcpp.12040 23347471 PMC3821531

[13] Carson C, Redshaw M, Gray R Risk of psychological distress in parents of preterm children in the first year: evidence from the UK Millennium Cohort Study. BMJ Open 2015;5:e007942 10.1136/bmjopen-2015-007942 PMC4691710 26685019

[14] Redshaw M, Henderson J Safely delivered: a national survey of women's experience of maternity care 2014. Oxford: NPEU, 2014.

[15] Green JM, Coupland VA, Kitzinger JV Expectations, experiences, and psychological outcomes of childbirth: a prospective study of 825 women. Birth 1990;17:15–24. 10.1111/j.1523-536X.1990.tb00004.x 2346576

[16] Padden T, Glenn S Maternal experiences of preterm birth and neonatal intensive care. J Reprod Infant Psychol 1997;15:121–39. 10.1080/02646839708404539

[17] Nicolaou M, Rosewell R, Marlow N Mothers’ experiences of interacting with their premature infants. J Reprod Infant Psychol 2009;27:182–94. 10.1080/02646830801922796

[18] Evans J, Heron J, Francomb H Cohort study of depressed mood during pregnancy and after childbirth. BMJ 2001;323:257–60. 10.1136/bmj.323.7307.257 11485953 PMC35345

[19] Evans J, Melotti R, Heron J The timing of maternal depressive symptoms and child cognitive development: a longitudinal study. J Child Psychol Psychiatry 2012;53:632–40. 10.1111/j.1469-7610.2011.02513.x 22211468

[20] Wolf MJ, Koldewijn K, Beelen A Neurobehavioral and developmental profile of very low birthweight preterm infants in early infancy. Acta Paediatr 2002;91:930–8. 10.1111/j.1651-2227.2002.tb02858.x 12222718

[21] Brazelton TB, Nugent JK The neonatal behavioural assessment scale. 4th edn Cambridge: MacKeith Press, 2011.

[22] Redshaw ME, Harris A Maternal perceptions of neonatal care. Acta Paediatr 1995;84:593–8. 10.1111/j.1651-2227.1995.tb13705.x 7670236

[23] Stern DN The first relationship: Infant and mother. 2nd edn Cambridge, MA: Harvard University Press, 2004.

[24] Nugent KJ, Keefer CH, Minear S Understanding Newborn Behavior and early relationships: the newborn behavioral observations (NBO) system handbook. Baltimore: Brookes Publishing Company, 2007.

[25] Redshaw M, Heikkila K Delivered with care: a national survey of women's experience of maternity care 2010. Oxford: NPEU, 2010.

[26] Care Quality Commission. National findings from the 2013 survey of women's experiences of maternity care. London: CQC, 2013.

[27] Leigh B, Milgrom J Risk factors for antenatal depression, postnatal depression and parenting stress. BMC Psychiatry 2008;8:24 10.1186/1471-244X-8-24 18412979 PMC2375874

[28] Bat-Erdene U, Metcalfe A, McDonald SW Validation of Canadian mothers’ recall of events in labour and delivery with electronic health records. BMC Pregnancy Childbirth 2013;13(Suppl 1):S3 10.1186/1471-2393-13-S1-S3 23445768 PMC3561172

[29] Quigley MA, Hockley C, Davidson LL Agreement between hospital records and maternal recall of mode of delivery: evidence from 12 391 deliveries in the UK Millennium Cohort Study. BJOG 2007;114:195–200. 10.1111/j.1471-0528.2006.01203.x 17166217

[30] Poulsen G, Kurinczuk JJ, Wolke D Accurate reporting of expected delivery date by mothers 9 months after birth. J Clin Epidemiol 2011;64:1444–50. 10.1016/j.jclinepi.2011.03.007 21684117

[31] Treyvaud K Parent and family outcomes following very preterm or very low birth weight birth: a review. Semin Fetal Neonatal Med 2014;19:131–5. 10.1016/j.siny.2013.10.008 24252709

